# Efficacy and safety of multiple doses of NEPA without dexamethasone in preventing nausea and vomiting induced by multiple-day and high-dose chemotherapy in patients with non-Hodgkin’s lymphoma undergoing autologous hematopoietic stem cell transplantation: a phase IIa, multicenter study

**DOI:** 10.1038/s41409-020-0909-2

**Published:** 2020-04-28

**Authors:** Nicola Di Renzo, Maurizio Musso, Rosanna Scimè, Alessandra Cupri, Tommasina Perrone, Clara De Risi, Domenico Pastore, Attilio Guarini, Andrea Mengarelli, Fabio Benedetti, Patrizio Mazza, Vera Capria, Patrizia Chiusolo, Luca Cupelli, Vincenzo Federico, Valentina Bozzoli, Anna Rita Messa, Paolo Codega, Erminio Bonizzoni, Giorgina Specchia

**Affiliations:** 1 Hematology and Transplant Unit, Vito Fazzi Hospital, Lecce, Italy; 2 Hematology and Transplant Unit, Oncology Department La Maddalena, Palermo, Italy; 3 UTMO Vito Cervello Hospital, Palermo, Italy; 4 Ferrarotto Hospital, Catania, Italy; 5 Hematology Unit, Department of Emergency and Organ Transplantation, University of Bari, Bari, Italy; 6 Hematology and Transplant Unit, Cardinal Panico Hospital, Tricase, Italy; 7 Hematology Unit, A. Perrino Hospital, Brindisi, Italy; 8 Hematology Unit, Giovanni Paolo II IRCCS Cancer Institute Oncology Hospital, Bari, Italy; 9 USOD Hematology and Transplant Unit, Regina Elena IFO National Cancer Institute, Roma, Italy; 10 Department of Medicine, Section of Hematology and Bone Marrow Transplant Unit, University of Verona, Verona, Italy; 11 Department of Hematology-Oncology, Moscati Hospital, Taranto, Italy; 12 Hematology Unit, Department of Translational and Precision Medicine, Policlinico Umberto I, Sapienza University, Roma, Italy; 13 Hematology Department, Fondazione Policlinico Universitario Agostino Gemelli, Roma, Italy; 14 Hematology Unit, Sant’Eugenio Hospital, Roma, Italy; 15 Medical Affairs Department, Italfarmaco SpA, Milan, Italy; 16 Section of Medical Statistics and Biometry GA Maccacaro, Department of Clinical Science and Community, University of Milan, Milan, Italy

**Keywords:** Phase II trials, Non-hodgkin lymphoma

## Abstract

Despite the availability of several antiemetics, clinical findings show that control of chemotherapy-induced nausea and vomiting (CINV) continues to be a serious concern for hematological patients, mainly for those receiving multiple-day (MD) and high-dose (HD) chemotherapy (CT). For CINV prophylaxis, 5-hydroxytryptamine type-3 receptor antagonists (5HT_3_-RAs) and neurokinin 1 receptor antagonists (NK_1_-RAs) are usually administered together with dexamethasone, which may increase the risk of serious infections in patients undergoing myeloablative treatment. The rationale of this multicenter, open-label and phase IIa study was to explore the efficacy of multiple doses of NEPA (netupitant/palonosetron) given as an every-other-day regimen without dexamethasone in preventing CINV in patients with relapsed-refractory aggressive non-Hodgkin’s lymphoma (R/R-NHL), eligible for autologous stem cell transplantation (ASCT) and treated with MD-HD-CT. Seventy patients participated to the study. According to the adopted Fleming one-stage design, the primary endpoint of this study was achieved. The CR values were 87.1% (primary endpoint, overall phase: days 1–8), 88.6% (acute phase: days 1–6), and 98.6% (delayed phase: days 7–8), while complete control (CR with no more than mild nausea) was 85.7% (overall phase), 88.6% (acute phase), and 95.7% (delayed phase). Moderate and severe episodes of nausea were reported by less than 10% of patients in the overall phase and less than 5% in both the acute and delayed phases. Regarding safety, NEPA was well tolerated with only one adverse event (constipation) evaluated as possibly related to NEPA administration. In conclusion, our study demonstrated that multiple alternate dosing of NEPA without the addition of dexamethasone is highly effective for preventing nausea and vomiting in this difficult setting, with a good tolerability profile.

## Introduction

Cancer chemotherapy (CT) may induce nausea and vomiting (CINV) and uncontrolled CINV can be detrimental for the patient, affecting the quality of life by causing dehydration, electrolyte imbalance, and malnutrition and therefore potentially altering patient adherence to life-saving treatments [1]. CINV may occur within 24 h after the start of CT (acute CINV), in the following days after the treatment (delayed CINV), or before CT administration (anticipatory CINV) [2, 3]. Anticipatory CINV has been correlated to the severity and duration of previous CT-associated emesis and therefore an effective prevention of CINV is highly recommended, especially in heavily pretreated patients [3–6].

Different factors concur in the development of CINV, some related to the treatment and some to the patient [7]. Among the antiemetics available for CINV prophylaxis, the most effective drugs are inhibitors of two receptors involved in the control of nausea and vomiting: 5-hydroxytryptamine type-3 receptor antagonists (5HT_3_-RAs) and neurokinin 1/substance P receptor antagonists (NK_1_-RAs). A combination of these two classes of molecules, together with dexamethasone, is recommended in all international guidelines for highly emetogenic CT (HEC) regimens [5, 6, 8]. NEPA is a fixed dose combination antiemetic that combines palonosetron, a second-generation 5HT_3_-RA, with a prolonged half-life and a higher receptor affinity compared with other first generation 5HT_3_-RAs [9–12], with netupitant a novel, highly selective NK_1_-RA [13, 14]. Both molecules have an extended half-life (palonosetron: 40 h; netupitant: 90 h) and their combination makes a single oral administration sufficient to cover the acute and the delayed phase of CINV induced by a single day CT [15–17]. NEPA plus dexamethasone showed superiority over oral palonosetron plus dexamethasone for all key efficacy endpoints during both the delayed and the overall (5 days) phases following either cisplatin-based HEC or anthracycline/cyclophosphamide CT [15, 16].

High-dose (HD) multiple-day (MD) CT and autologous stem cell transplant support represent the potential curative strategy for most hematological malignancies. Although the international guidelines recommend the three-drug combination 5HT_3_-RA/NK_1_-RA/dexamethasone for patients treated with HD-CT and stem cell or bone marrow transplantation, CINV management remains a significant problem in this setting of patients [5, 6]. Some studies have been conducted with different 5HT_3_-RAs in combination with aprepitant (NK_1_-RA) and dexamethasone using different schedules [18–21]. However, trials investigating the efficacy and the safety of NEPA in hematological settings are not yet available. Notably, the studies were performed using dexamethasone that contributes to CINV prophylaxis by its intrinsic antiemetic properties and by interacting with 5HT_3_-RAs/NK_1_-RAs [22] but that also exhibits an important immunosuppressive activity, which could lead to several adverse events, such as increasing the risk of serious infections, especially in patients undergoing hematopoietic stem cell transplantation.

The rationale of this study was to explore the efficacy and the safety of multiple doses of NEPA given as an every-other-day regimen without dexamethasone in preventing CINV in patients with non-Hodgkin’s lymphoma (NHL), eligible for autologous stem cell transplantation (ASCT) and treated with MD-HD-CT, such as FEAM/BEAM regimen.

## Subjects and methods

### Study design

This trial was planned as a phase IIa, open-label, noncomparative study with a one-stage Fleming design and conducted between January 2016 and February 2018 in 28 Italian centers. Each center obtained approval from the local institutional review board/ethics committee and a written informed consent was collected from each patient before study enrollment.

### Patients

Patients were eligible for the study if ≥18-years old and with a diagnosis of relapsed/refractory aggressive NHL eligible for ASCT. After enrollment, the patients underwent the mobilization phase consisting of MD-CT with granulocyte-colony stimulating factor support and HSC collection by leukapheresis.

After HSC collection, if the eligibility for ASCT was confirmed, the patients entered the study period consisting of the conditioning phase. The conditioning regimen was either BEAM (carmustine 300 mg/m^2^ on day −6, etoposide 200 mg/m^2^, and cytarabine 400 mg/m^2^ on days −5, −4, −3, and −2, and melphalan 140 mg/m^2^ on day −1) or FEAM (fotemustine 300 mg/m^2^ on day −6, etoposide 200 mg/m^2^, and cytarabine 400 mg/m^2^ on days −5, −4, −3, and −2, and melphalan 140 mg/m^2^ on day −1). Day 0 was the day of ASCT. NEPA were administered every other day of HD-MD-CT administration, starting from the first day of carmustine or fotemustine administration (day −6 before ASCT) with the second and the third dose of NEPA given after 2 and 4 days (days −4 and day −2), respectively.

Use of dexamethasone for antiemetic prophylaxis was not allowed, in order to decrease the risk of serious infections in these patients who were already heavily immunosuppressed. In case of need, oral metoclopramide (max 30 mg/day) was used as rescue antiemetic. Treatment-emergent adverse events (TEAEs) were also monitored and recorded during the study according to the Common Terminology Criteria for Adverse Events ver 4.3.

### Assessment

Nausea and vomiting were monitored using a self-compiled study diary, in which each episode of emesis, any use of rescue medication, the maximum grade of nausea according to the Likert scale (none, mild, moderate, and severe), and occurrence of any adverse event were recorded daily from day 1 of CT until day 15 throughout the study period (overall phase and follow-up). At the end of the observation period, the patients’ global satisfaction with the CINV prophylaxis was also collected by means of a visual analog scale.

### Study objectives

#### Primary endpoint

The primary endpoint was to evaluate the rate of complete response (no emesis and no rescue medication) after the administration of multiple doses of NEPA during the overall period of the conditioning phase (study period), defined from day 1 until 2 days after the last dose of CT. According to the study design and sample size assumptions, the primary endpoint was defined according to the one-tailed statistical hypotheses: null hypothesis (H0): π < π_0_ = 0.50/alternative hypothesis (H1): π ≥ π_1_ = 0.65. Therefore, with 41 or less complete responders out of 69 patients, the activity was shown to be less than desired, while with 42 or more responders, there was some activity and so the primary endpoint was shown to be achieved; where π, π_0_, and π_1_ were defined as the “observed response rate”, the “maximum response rate of a poor treatment”, and the “minimum response rate of a good treatment” respectively. Sample size computations were carried out using the PASS 14 Software.

#### Secondary endpoints

Secondary endpoints were to evaluate complete response (no emesis, no rescue medication), complete control (complete response with a maximum grade of mild nausea), emesis-free (no emesis), rescue-free (no rescue medication), nausea, and patient global satisfaction rates with antiemetic therapy in the acute phase (during days of CT administration), the delayed phase (48 h after the last dose of CT), the overall phase, and on each day of the CT.

## Results

**Fig. 1:**
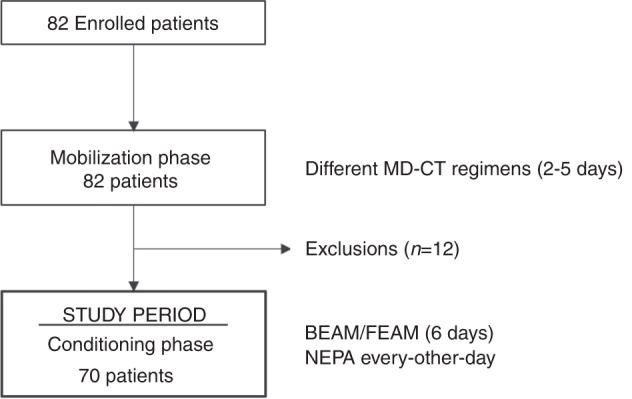
Study flow diagram. Before entering the study period, enrolled patients underwent the mobilization phase. Then, patients were reassessed for eligibility and if positive entered the study, corresponding to the conditioning phase, where NEPA was administered every other day for CINV prophylaxis.

Among the 82 patients that were enrolled in the study and underwent to the mobilization phase, 70 patients were eligible for the conditioning phase and ASCT and therefore participated in the study period (see Fig. 1 and Table 1). Reasons for noneligibility were seven changes of treatment (six disease progressions, one remission), two failed stem cell mobilization, one sepsis, one allogeneic transplantation, and one death.

**Table 1:** Summary of patients’ characteristics.

Age (years)	Mean ± SD (N)	54.04 ± 10.77 (82)
	Median (25th–75th)	56.5 (48–62)
	Min–Max	24–78
Gender	Female	31 (37.8%)
	Male	51 (62.2%)
ECOG	0	48 (58.5%)
	1	29 (35.4%)
	2	5 (6.1%)
Mobilization Regimen	DHAOX	3 (3.7%)
	DHAP	29 (35.4%)
	GIFOX	1 (1.2%)
	IEV	16 (19.5%)
	IGEV	1 (1.2%)
	IVAC	1 (1.2%)
	R-DHAOX	5 (6.1%)
	R-DHAP	20 (24.4%)
	R-EPOCH	1 (1.2%)
	R-ICE	2 (2.4%)
	R-IEV	2 (2.4%)
	R-MTX ARAC HD	1 (1.2%)
Duration of mobilization regimen	2 days	58 (70.7%)
	3 days	21 (25.6%)
	4 days	1 (1.2%)
	5 days	2 (2.4%)
Conditioning regimen	BEAM	23 (28%)
	FEAM	46 (56.1%)
	Melphalan/Mitoxatrone	1 (1.2%)
	None	12 (14.6%)

The table reports age, gender, the ECOG performance status, and the chemotherapy regimen administered of all 82 patients enrolled in the study.

**Fig. 2:**
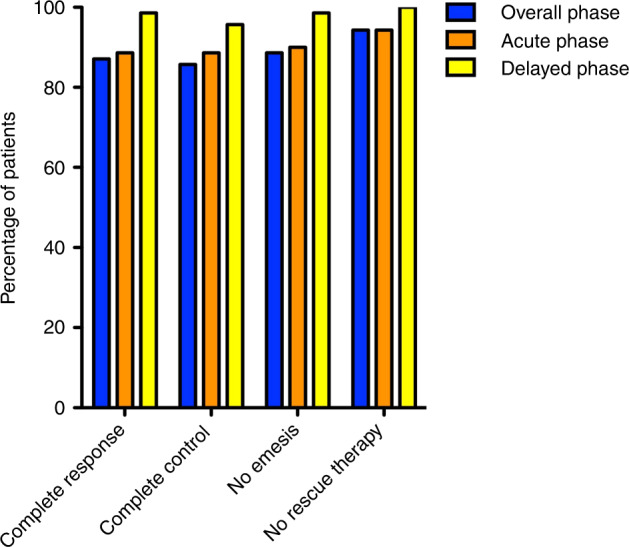
Patient responders. The histograms show the patients’ percentages for the acute (days 1–6), delayed (days 7–8) and overall (days 0–8) phases classified as complete response (no emesis, no rescue medication), complete control (complete response and no more than mild nausea), no emesis, and no rescue therapy.

**Fig. 3:**
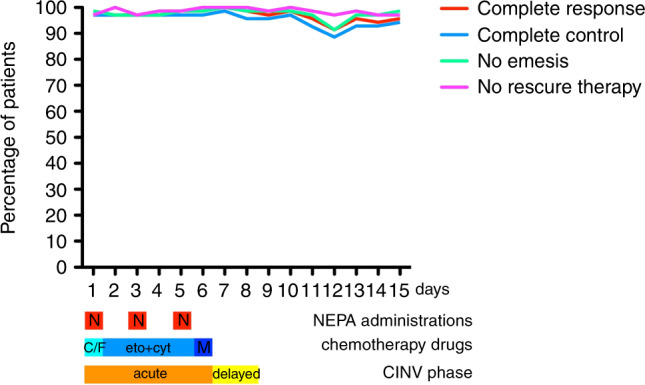
Patient responders—daily recordings. The line graph shows patients’ percentage classified as complete response (no emesis, no rescue medication), complete control (complete response and no more than mild nausea), no emesis, and no rescue therapy monitored for 15 days during and following the BEAM/FEAM regimens. NEPA (N) was given on days 1, 3, and 5. Carmustine (C) or fotemustine (F) was administered on day 1, etoposide (eto) and cytarabine (cyt) from days 2 to 5, and melphalan (M) on day 6.

**Fig. 4:**
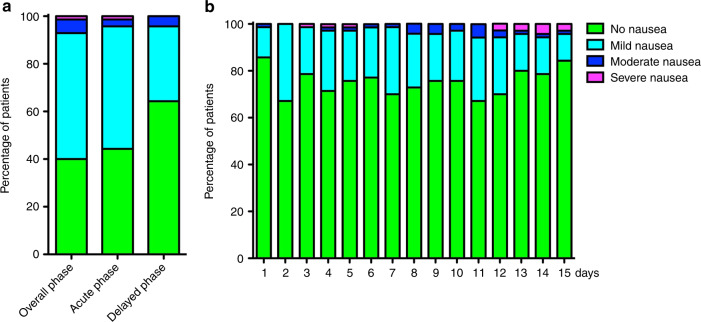
Nausea. **a** The histograms show the patients’ percentages for the acute (days 1–6), delayed (days 7–8), and overall (days 0–8) phases according to their level of nausea (none, mild, moderate, and severe). **b** The histograms show nausea levels monitored for 15 days during and following the BEAM/FEAM regimens.

According to the Fleming one-stage study design, the primary endpoint of this study was achieved. Indeed, the number of complete responders in the overall phase among the first consecutive 69 patients of the study was 60, which is greater than the fixed cutoff of 42, representing the minimum number of responders for which the treatment is considered effective. In addition to the primary endpoint, several additional endpoints were evaluated and all demonstrated the efficacy of NEPA in this setting (Fig. 2). Indeed, the CR values were 87.1% (primary endpoint, overall phase), 88.6% (acute phase), and 98.6% (delayed phase), while the complete control (CR with no more than mild nausea) was 85.7% (overall phase), 88.6% (acute phase), and 95.7% (delayed phase). Moreover, the percentages of patients that did not suffer any emetic episodes were 88.6% (overall phase), 90% (acute phase), and 98.6% (delayed phase), and of patients that did not require a rescue therapy for controlling CINV were 94.3% (overall phase), 94.3% (acute phase), and 100% (delayed phase). Daily values for each of these categories were recorded during the 8 days of the study and the other 7 days of follow-up (Fig. 3). Similarly, patients also documented the grade of their nausea according to the Likert scale. Moderate and severe episodes of nausea were reported by less than 10% in the overall phase and less than 5% in both acute and delayed phases and the daily values of no nausea were above 65% for each day of the treatment (Fig. 4a, b). Indeed, the mean patient global satisfaction for the antiemetic prophylaxis for the study period was 9.13 ± 1.59 out of 10.

Regarding the safety profile, NEPA was very well tolerated, with only 12 TEAEs that occurred in six (8.6%) subjects during the study. Among these, only one event of constipation was evaluated as possibly related to NEPA administration. Incidences of TEAEs were quite low apart from fever, which occurred with an incidence > 5%. Only one TEAE (fever) was graded as severe, whilst two TEAEs (both fever) were classified as serious adverse event (SAE). All the TEAEs graded as severe or classified as SAE have been evaluated as not related to NEPA administration.

## Discussion

Proper management of CINV remains a challenge in hematological malignancies. A large number of patients are intensively treated, and many are at particularly high risk of CINV due to a high prevalence of CINV risk factors. Indeed, hematological patients are often young and are often treated with MD and/or HD CT regimens [23, 24]. Despite clinical findings in CINV prophylaxis, control of nausea and vomiting continues to be a significant problem particularly in the days after the start of CT since MD-HD regimens trigger a prolonged, enhanced, and diverse emetogenic stimulus during each day of the treatment [25, 26]. Indeed, studies have evaluated that CINV prophylaxis based on palonosetron and dexamethasone, either in single or multiple dose, do not provide an extensive control of nausea and vomiting in patients undergoing MD-CT and MD-HD-CT [27, 28]. Similar rates were found in trials that evaluated the efficacy of the triple combination 5HT_3_-RA/NK_1_-RA/dexamethasone using ondansetron or tropisetron and aprepitant in patients treated for several days with ablative preparative regimens [18, 20, 21]. However, proper comparisons are hampered by the lack of comparable endpoints and discrepancies in schedules, including inconsistent length of acute/delayed phases, different drug administrations, and distinct usage of corticosteroids.

In our study, the administration of NEPA with an every-other-day schedule demonstrated an excellent CINV control reaching a CR rate of 87.1% in the overall phase, defined as no emetic episodes and no rescue therapy. NEPA-based prophylaxis resulted not only very effective in the prevention of emesis but also quite efficient in controlling nausea. Indeed, during the overall phase the percentage of patients experiencing no more than mild nausea was 92.9% with daily values of no nausea well above 65% for all the days of CT and the following 10 days. For the same CT regimen (BEAM), a CINV prophylaxis based on palonosetron or ondansetron, aprepitant and high dose of dexamethasone (20 mg on the first day followed by 12 mg daily) showed a response rate defined as “highly effective” in 82% of the patients; this definition includes patients that did not experience vomiting and had no more than mild nausea and patients that had 1–2 emetic episodes and no nausea [21]. In comparison, the results obtained in our study are highly relevant being more effective and achieved without the use of HD dexamethasone. Despite dexamethasone is generally considered a safe drug andis recommended by the antiemetic guidelines in combination with 5HT_3_-RAs and NK_1_-RAs, its administration may be associated with a wide range of side effects [29, 30], including additional immunosuppression that can lead to immunological complications in severely debilitated patients undergoing several lines of CT and myeloablative treatments. Some encouraging studies that investigate dexamethasone-sparing antiemetic prophylaxes were already conducted in hematology settings with palonosetron alone [31, 32]. However, this is the first study that evaluates the efficacy of an antiemetic prophylaxis in the transplantation setting without the use of dexamethasone as antiemetic or as part of the CT regimen.

The use of palonosetron with an every-other-day schedule has already been investigated and established [28] and this study aimed also to confirm the safety profile of netupitant administered on multiple days. Moreover, netupitant is a moderate inhibitor of cytochrome P450 3A4 (CYP3A4) and it may interfere with the pharmacokinetics of other drugs that interact with the same enzyme. Indeed, it alters the clearance of etoposide, the only BEAM/FEAM drug that is a CYP3A4 substrate [33]. In our study, multiple doses of NEPA were well tolerated, and no SAEs were detected by an increased exposure to netupitant. Moreover, our study confirms the absence of clinical impact due to an extended bioavailability of etoposide and thus can be considered safe for use with BEAM/FEAM regimens.

Overall, the administration of every-other-day NEPA was found to be very effective in controlling both emesis and nausea in patients at high risk of CINV undergoing FEAM/BEAM-based MD-HD-CT. This approach combines the most effective antiemetic prophylaxis with a simplification of the therapy, which also spares the use of corticosteroids in these heavily pretreated and immunocompromised patients.
